# Association of Sick Sinus Syndrome with Incident Cardiovascular Disease and Mortality: The Atherosclerosis Risk in Communities Study and Cardiovascular Health Study

**DOI:** 10.1371/journal.pone.0109662

**Published:** 2014-10-06

**Authors:** Alvaro Alonso, Paul N. Jensen, Faye L. Lopez, Lin Y. Chen, Bruce M. Psaty, Aaron R. Folsom, Susan R. Heckbert

**Affiliations:** 1 Division of Epidemiology and Community Health, School of Public Health, University of Minnesota, Minneapolis, Minnesota, United States of America; 2 Department of Epidemiology, School of Public Health, University of Washington, Seattle, Washington, United States of America; 3 Cardiovascular Division, Department of Medicine, University of Minnesota Medical School, Minneapolis, Minnesota, United States of America; 4 Cardiovascular Health Research Unit, Departments of Medicine, Epidemiology, and Health Services, University of Washington, Seattle, Washington, United States of America; 5 Group Health Research Institute, Group Health Cooperative, Seattle, Washington, United States of America; University of Bologna, Italy

## Abstract

**Background:**

Sick sinus syndrome (SSS) is a common indication for pacemaker implantation. Limited information exists on the association of sick sinus syndrome (SSS) with mortality and cardiovascular disease (CVD) in the general population.

**Methods:**

We studied 19,893 men and women age 45 and older in the Atherosclerosis Risk in Communities (ARIC) study and the Cardiovascular Health Study (CHS), two community-based cohorts, who were without a pacemaker or atrial fibrillation (AF) at baseline. Incident SSS cases were validated by review of medical charts. Incident CVD and mortality were ascertained using standardized protocols. Multivariable Cox models were used to estimate the association of incident SSS with selected outcomes.

**Results:**

During a mean follow-up of 17 years, 213 incident SSS events were identified and validated (incidence, 0.6 events per 1,000 person-years). After adjustment for confounders, SSS incidence was associated with increased mortality (hazard ratio [HR] 1.39, 95% confidence interval [CI] 1.14–1.70), coronary heart disease (HR 1.72, 95%CI 1.11–2.66), heart failure (HR 2.87, 95%CI 2.17–3.80), stroke (HR 1.56, 95%CI 0.99–2.46), AF (HR 5.75, 95%CI 4.43–7.46), and pacemaker implantation (HR 53.7, 95%CI 42.9–67.2). After additional adjustment for other incident CVD during follow-up, SSS was no longer associated with increased mortality, coronary heart disease, or stroke, but remained associated with higher risk of heart failure (HR 2.00, 95%CI 1.51–2.66), AF (HR 4.25, 95%CI 3.28–5.51), and pacemaker implantation (HR 25.2, 95%CI 19.8–32.1).

**Conclusion:**

Individuals who develop SSS are at increased risk of death and CVD. The mechanisms underlying these associations warrant further investigation.

## Introduction

Sick sinus syndrome (SSS) is a disorder characterized by symptomatic dysfunction of the sinoatrial node. On the electrocardiogram (ECG), SSS usually manifests as sinus bradycardia, sinus arrest, or sinoatrial block, sometimes accompanied by supraventricular tachyarrhythmias (“tachy-brady” syndrome). Typical symptoms of SSS include syncope, dizziness, palpitations, exertional dyspnea and easy fatigability from chronotropic incompetence, heart failure, or angina [1]–[3]. Recent estimates suggest that>75,000 new cases of SSS occur in the US every year and that this number will more than double by 2060 [4].

Despite being relatively frequent and a major indication for pacemaker implantation [5], the impact of SSS on the risk of other cardiovascular outcomes and mortality has received little attention. The existing evidence on this issue is limited to clinical series and randomized trials of pacemaker implantation [6]–[11]. Overall, these studies show that mortality in patients with SSS can be substantial and potentially explained by the presence of other comorbidities. Most of these reports, however, did not directly compare outcomes in patients with SSS to individuals without this condition and, thus, the association of SSS occurrence with overall survival and risk of cardiovascular disease (CVD) in the general population, independent of other risk factors, remains unclear.

With the general aim of providing current and valid information on the prognosis of SSS, we identified and validated SSS events, and evaluated whether incident SSS was associated with mortality and CVD events in two large community-based cohorts, the Atherosclerosis Risk in Communities (ARIC) study and the Cardiovascular Health Study (CHS).

## Methods

### Study population

In 1987–89, the ARIC study recruited 15,792 men and women aged 45–64 from 4 US communities (Forsyth Co, NC; Jackson, MS; Minneapolis suburbs, MN; and Washington Co, MD) with the aim of identifying risk factors of atherosclerosis and incidence of CVD in the general population [12]. Participants were mostly white in the Minneapolis and Washington Co field centers, both white and black in Forsyth Co, while only blacks were recruited in Jackson. Study participants completed 4 follow-up visits in 1990–1992, 1993–1995, 1996–1998, and 2011–2013. Since baseline, participants have been called annually to obtain information on vital status, hospitalizations, and occurrence of CVD (response rate>90%).

Between 1989 and 1990, CHS recruited 5201 men and women aged 65 and older selected from Medicare eligibility lists in 4 US counties (Allegheny, PA; Forsyth, NC; Sacramento, CA; Washington, MD). An additional 687 participants, nearly all blacks, were recruited in 1990–1992 [13], [14]. Participants had annual exams through 1999, with a follow-up phone call between exams. Since 1999, participants have been contacted semi-annually on the phone to determine vital status, hospitalizations, and occurrence of CVD. Average response rate to follow-up annual contacts was 92%.

For this analysis, we excluded participants who met any of the following criteria at baseline: atrial fibrillation (AF) or presence of a pacemaker, heart rate <50 bpm while not using beta-blockers, missing ECG data or covariates, or, in the ARIC study, individuals not reporting white or black race, as well as blacks in the Minneapolis and Washington County field centers (because of small numbers). After exclusions, 14,816 individuals in ARIC and 5077 in CHS were included.

All study participants provided written informed consent at baseline and follow-up exams. The University of Minnesota Institutional Review Board and the University of Washington Institutional Review Board approved the present study.

### Ascertainment of SSS

Details of SSS ascertainment and validation have been published elsewhere [4]. Briefly, hospitalizations during follow-up were identified through follow-up telephone calls, surveillance of local hospitals (only in ARIC), and inpatient Medicare claims (only in CHS). In both studies, trained abstractors collected information on all hospitalizations. Possible SSS cases were identified if a hospitalization listed International Classification of Diseases (ICD) 9 code 427.81 [sick sinus syndrome, sinus node dysfunction, tachy-brady syndrome] among the discharge diagnoses. Available medical records were reviewed by at least one study investigator. SSS was considered to be present if the record included a medical diagnosis of SSS and symptoms or signs consistent with SSS (e.g. syncope, dizziness, bradycardia, sinus pauses), without evidence of other conditions responsible for the episode, such as atrioventricular block or medication use. In the ARIC study, 294 individuals had an ICD9 code 427.81 in at least 1 hospitalization. Medical charts at the ARIC study sites were available in 195, and 130 were confirmed as SSS after review (no additional efforts were made to retrieve missing records from hospitals). Of these, 117 occurred among eligible participants. In CHS, the ICD9 code 427.81 was present in 179 individuals and a SSS diagnosis was confirmed in 99 of the 169 individuals with available medical charts, 96 of them in eligible participants. Only confirmed SSS cases were included in the primary analysis.

### Outcome ascertainment

The outcomes of interest included coronary heart disease (CHD), stroke, heart failure, atrial fibrillation, pacemaker implantation, and all-cause mortality occurring during the follow-up. In both the ARIC study and CHS, incident CHD and stroke were validated using established criteria with physician review of records [15]–[17]. Specifically, incident CHD was defined as definite or probable myocardial infarction, or definite coronary death. Angina and subclinical CHD were not included in this definition. Stroke (ischemic or hemorrhagic) was defined as the sudden or rapid onset of neurological symptoms lasting for at least 24 hours or leading to death in the absence of evidence for a non-stroke cause. Incident heart failure in the ARIC study was defined as presence of an ICD code for heart failure (ICD9 428, ICD10 I50) in a hospitalization or death certificate [18]. In CHS, heart failure events were adjudicated based on review of study exams and hospital records [17]. Incident AF was identified from ECGs done during study exams, presence of ICD9 codes 427.31 or 427.32 in any hospitalization, from death certificates including AF as a cause of death (in ARIC only; ICD9 427.3 or ICD10 I48), and when 2 outpatient ICD9 codes 427.31 or 427.32 were present in Medicare claims within a 1-year period (in CHS only) [19]–[21]. Pacemaker implantation was based on the presence of the following ICD9 codes in any hospitalization: 37.8 (insertion, placement and revision of pacemaker), V45.01 (status post-pacemaker implantation), or V53.31 (fitting and adjustment of cardiac pacemaker). For pacemaker implantation occurring in the same hospitalization as the SSS diagnosis, we defined the length of follow-up as one day. Finally, information on all-cause mortality was obtained from follow-up calls, review of obituaries in local newspapers, and linkage to the National Death Index.

### Assessment of other covariates

Measurements followed similar protocols in both ARIC and CHS. At baseline, study participants provided information on education, smoking status, medication use, and prevalence of cardiovascular disease. A physical examination collected information on height and weight, and blood pressure was measured with a random-zero sphygmomanometer. Fasting glucose and lipids were measured from blood samples obtained during the exam. Diabetes was defined as a fasting glucose level of ≥126 mg/dl, a non-fasting glucose of ≥200 mg/dl, use of an oral hypoglycemic agent or insulin, or, in the ARIC study, self-reported medical diagnosis of diabetes.

### Statistical analysis

We calculated age-, sex- and race-specific incidence rates of the different outcomes among those with and without SSS in ARIC and CHS. Person-time for incidence calculations in those with SSS started at the time of SSS diagnosis until the outcome of interest, death, or censoring (December 31, 2009 in ARIC, or June 30, 2008 in CHS). Person-time for event rates during follow-up without SSS was calculated in a similar way, starting at baseline until SSS incidence, incidence of the outcome of interest, death, or censoring. Age, sex, and race-standardized rates were calculated via direct standardization using the pooled ARIC and CHS person-time as the reference. The associations between SSS incidence and each of the outcomes were assessed with Cox proportional hazards model including incident SSS as a time-varying exposure and adjusting for baseline covariates. Separate models were run in each cohort for the different outcomes. Initial models (Model 1) adjusted for age, sex, race, study center, education, smoking, body mass index, hypertension, total cholesterol, HDL cholesterol, diabetes, prevalent CHD, prevalent heart failure, and prevalent stroke. Additional models (Model 2) also adjusted for nonfatal incident CHD, incident HF, incident stroke and incident AF as time-dependent covariates, excluding the corresponding time-dependent incident disease from models in which that specific disease was the outcome. Individuals with prevalent stroke, CHD or heart failure at baseline were excluded from the corresponding analysis of incident disease. To address the impact of pacemaker implantation in the risk of AF and HF in those with SSS, we conducted two additional analyses. First, we included pacemaker implantation as a time-dependent covariate. Second, in a separate analysis, we subclassified SSS as SSS with a pacemaker and SSS without pacemaker.

Cohort-specific results were meta-analyzed to provide pooled hazard ratios using inverse-of-variance weighting. Between-cohort heterogeneity was assessed with Cochran's Q and I^2^ statistics [22]. Because medical records were not available for one third of cases with ICD9 427.81 in the ARIC study, and therefore could not be validated, we conducted a sensitivity analysis in the ARIC cohort including these possible SSS cases (n = 82) in addition to the validated cases. Analyses were conducted using SAS 9.3 (SAS Institute, Cary, NC) and Stata 12 (Stata Corp, College Station, TX).

## Results

Among 19,893 eligible participants (14,816 in ARIC, 5077 in CHS), 213 incident SSS events were identified and validated (117 in ARIC, 96 in CHS) during a mean follow up of 17 years. The crude incidence rate of confirmed SSS was 0.4 and 1.5 per 1000 person-years in ARIC and CHS, respectively. In both cohorts, participants who developed SSS had a higher body mass index, were more likely to be white, and had higher prevalence of hypertension, diabetes, heart failure and CHD, compared to those without SSS (Table 1).

**Table 1:** Baseline characteristics by sick sinus syndrome (SSS) diagnosis during follow-up, Atherosclerosis Risk in Communities (ARIC) study and Cardiovascular Health Study (CHS).

	ARIC		CHS	
	SSS	No SSS	SSS	No SSS
N	117	14699	96	4981
Age, years	57.7 (4.7)	54.1 (5.8)	72.3 (5.2)	72.7 (5.5)
Women, %	53	56	63	59
Non-white, %	13	26	7	16
Completed high school, %	77	77	69	71
Current smoker, %	21	26	8	12
BMI, kg/m^2^	29.2 (5.4)	27.7 (5.4)	27.7 (4.8)	26.7 (4.7)
Hypertension, %	47	35	68	58
Diabetes, %	21	12	18	16
Total cholesterol, mg/dL	218 (40)	215 (42)	213 (40)	212 (39)
HDL cholesterol, mg/dL	45.7 (14.3)	51.5 (17.1)	54.3 (15.6)	54.4 (15.9)
Prevalent CHD, %	8	5	20	18
Prevalent HF, %	8	5	5	4
Prevalent stroke, %	1	2	3	4

Values correspond to means (standard deviations) or proportions. BMI: body mass index; CHD: coronary heart disease; HF: heart failure.

**Figure 1:**
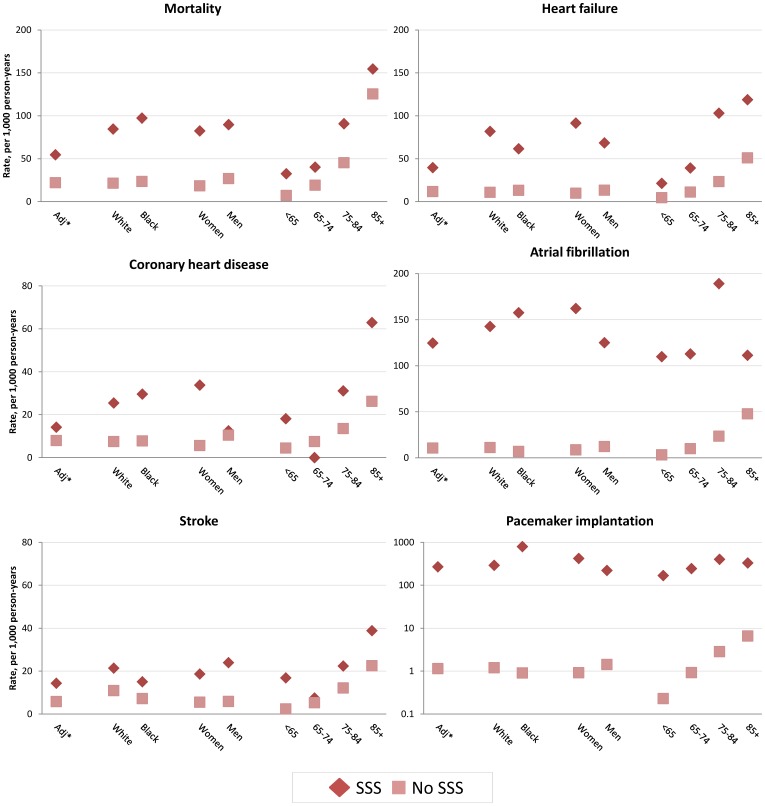
Incidence rates of selected cardiovascular diseases by SSS status, overall and by race, sex and age groups, combined Atherosclerosis Risk in Communities study and Cardiovascular Health Study, 1987–2009. Dark squares correspond to rates in SSS, light diamonds to rates in non SSS. Adj*: Standardized by age, sex and race to the combined ARIC and CHS person-time.

Overall, individuals with SSS had higher age, race, and sex-standardized incidence rates of mortality and CVD than those without SSS (Table 2). Standardized mortality rates were 55 per 1000 person-years in those with SSS compared to 22 per 1000 person-years in those without SSS (rate ratio 2.5, 95% confidence interval (CI) 1.4–4.3). Similar rate ratios were observed for heart failure, CHD, and stroke, while rates of AF and pacemaker implantation were more than 10 and 200 times higher for those with SSS compared to those without SSS, respectively. SSS was associated with higher mortality and CVD rates in men and women, whites and non-whites, and across age groups (Figure 1).

**Table 2:** Age, race, and sex-standardized rates (per 1000 person-years) of mortality and selected cardiovascular events in individuals with and without sick sinus syndrome (SSS), combined Atherosclerosis Risk in Communities study, 1987–2009, and Cardiovascular Health Study, 1989–2008.

	No SSS			SSS			IRR (95% CI)
	Cases	Person-years	Rate (95%CI)	Cases	Person-years	Rate (95%CI)	
Mortality	7471	341,775	22.0 (21.5–22.5)	97	1135	54.5 (24.6–84.5)	2.5 (1.4–4.3)
Coronary heart disease	2326	309,467	7.9 (7.6–8.2)	22	854	14.2 (0.0–28.1)	1.8 (0.7–4.8)
Stroke	1831	327,396	5.7 (5.5–6.0)	20	955	14.3 (2.7–25.9)	2.5 (1.1–5.6)
Heart failure	3502	311,658	11.6 (11.2–12.0)	54	672	39.5 (22.7–56.3)	3.4 (2.2–5.2)
Atrial fibrillation	3256	327,078	10.5 (10.2–10.8)	60	416	125 (76.2–173)	12 (8.1–18)
Pacemaker implantation	423	340,162	1.1 (1.0–1.3)	132	423	268 (199–338)	235 (178–310)

CI: confidence interval; IRR: incidence rate ratio.

**Figure 2:**
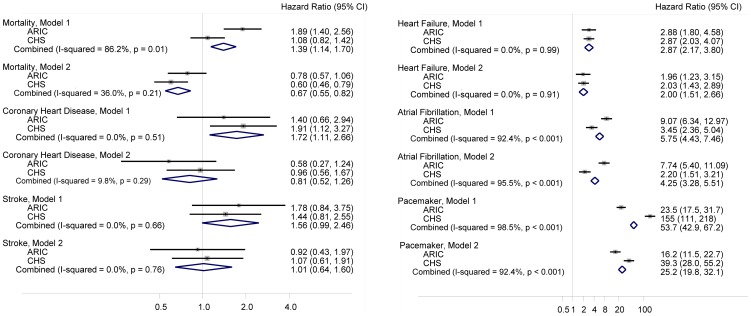
Cohort-specific and pooled hazard ratios (95% confidence intervals) of mortality and selected cardiovascular diseases comparing individuals with and without sick sinus syndrome (SSS), Atherosclerosis Risk in Communities (ARIC) study, 1987–2009, and Cardiovascular Health Study (CHS), 1989–2008. Model 1: Cox proportional hazards model adjusted for age, sex, race, study center, education, smoking, body mass index, hypertension, total cholesterol, HDL cholesterol, diabetes, prevalent coronary heart disease, prevalent heart failure, and prevalent stroke. Model 2: Adjusted as in model 1, and for incident coronary heart disease, incident heart failure, incident stroke and incident atrial fibrillation as time-dependent covariates.

SSS was associated with a higher incidence of all outcomes after adjustment for baseline covariates (including sociodemographic and clinical factors), with hazard ratios ranging from 1.4–1.7 for mortality, stroke and CHD, to 2.9 for heart failure, 5.8 for AF, and 54 for pacemaker implantation (Figure 2, Model 1). The higher risk of AF, heart failure and pacemaker implantation associated with SSS remained after adjusting for incident of other CVD as a time-dependent variable, though increased risk of death and other CVD among participants with SSS no longer were present (Figure 2, Model 2). Analysis restricted to the ARIC cohort that incorporated possible SSS events without available charts in addition to validated cases yielded similar results (Table S1).

Adjustment for pacemaker implantation as a time-dependent covariate attenuated, but did not eliminate, the association of SSS with increased risk of AF and HF (Table 3). In an additional analysis classifying SSS patients according to pacemaker implantation status, we found that the elevated risk of AF and HF in those with SSS was lower among those with a pacemaker than among those without one (Table 3).

**Table 3:** Cohort-specific and pooled hazard ratios (95% confidence intervals) for the association of sick sinus syndrome (SSS) with atrial fibrillation and heart failure, adjusting for cardiovascular risk factors and accounting for pacemaker implantation, Atherosclerosis Risk in Communities (ARIC) study, 1987–2009, and Cardiovascular Health Study (CHS), 1989–2008.

Adjustment for pacemaker implantation as a time-dependent covariate				
		ARIC	CHS	Pooled
Heart failure	No SSS	1 (ref.)	1 (ref.)	1 (ref.)
	SSS^a^	1.55 (0.91–2.65)	1.99 (1.30–3.06)	1.80 (1.29–2.52)
Atrial fibrillation	No SSS	1 (ref.)	1 (ref.)	1 (ref.)
	SSS^a^	5.39 (3.56–8.14)	2.15 (1.42–3.26)	3.40 (2.55–4.57)

Categorization of SSS status by pacemaker implantation				
Heart failure	No SSS	1 (ref.)	1 (ref.)	1 (ref.)
	SSS without pacemaker^b^	2.35 (1.22–4.54)	3.12 (1.85–5.25)	2.80 (1.86–4.21)
	SSS with pacemaker^b^	1.68 (0.87–3.26)	1.60 (1.01–2.53)	1.63 (1.12–2.37)
Atrial fibrillation	No SSS	1 (ref.)	1 (ref.)	1 (ref.)
	SSS without pacemaker^b^	8.01 (4.77–13.4)	2.64 (1.68–4.15)	4.27 (3.03–6.00)
	SSS with pacemaker^b^	7.18 (4.36–11.8)	1.63 (0.84–3.16)	4.20 (2.82–6.26)

a,b Results correspond to Cox proportional hazards model adjusted for age, sex, race, center, education, smoking, BMI, hypertension, total cholesterol, HDL cholesterol, diabetes, prevalent and time-dependent CHD, prevalent and time-dependent HF (for the AF analysis only), prevalent and time-dependent stroke, time-dependent AF (for the HF analysis only). Model ^a^ additionally adjusted for time-dependent pacemaker implantation.

We observed significant between-cohort heterogeneity for the association between SSS and mortality, AF and pacemaker implantation. In the ARIC study, but not in CHS, SSS was associated with increased mortality after adjustment for cardiovascular risk factors. Similarly, the association of SSS with AF risk was stronger in the ARIC study than in CHS. In contrast, SSS was more strongly associated with pacemaker implantation in CHS than in ARIC (Figure 2).

## Discussion

In this analysis of two community-based cohort studies in the US, we observed a higher risk of mortality and incident CVD, particularly AF, in individuals with SSS compared with those without SSS. As expected, SSS occurrence was a strong predictor of pacemaker implantation. Associations between SSS and investigated outcomes were weakened after adjustment for baseline covariates and incident CVD occurring during the follow-up. Between-cohort heterogeneity was observed for some outcomes.

Few prior studies have assessed mortality and CVD risk among patients with SSS compared to unaffected individuals. Most current information derives from clinical series or pacemaker implantation trials. Two studies compared age- and sex-adjusted mortality rates in SSS patients with rates observed in the general population [6], [7]. In these studies, SSS patients without structural heart disease experienced mortality rates similar to those in the general population; however, this comparison can be problematic since the general population includes both healthy and sick individuals, potentially masking differences in mortality. Published clinical trials of pacemaker implantation modalities have offered information on mortality and CVD rates in SSS patients. These trials have consistently reported high mortality rates among patients with SSS (in excess of 5%/year), and considerable incidence of AF and other cardiovascular complications [8]–[11], which may be explained by the high prevalence of cardiovascular risk factors among SSS patients [23]–[25]. Trials, however, do not clarify the association of SSS with mortality and CVD compared to individuals without the condition. To the best of our knowledge, our analysis is the first to make this comparison in the general population controlling for cardiovascular risk factors and other potential confounding variables.

We found that individuals with SSS had higher mortality, stroke, and CHD rates than those without SSS. These differences disappeared after adjusting for cardiovascular risk factors and incident CVD. In contrast, the risk of heart failure and AF remained elevated in SSS patients compared to non-SSS individuals even after multiple adjustments. Taken together, our results would suggest that SSS patients have elevated mortality and higher risk of stroke and CHD in part through the association of SSS with higher incidence of AF and heart failure. CHD is an established risk factor for AF [26], [27] and potentially for SSS [4], [28], but recent findings suggest that AF also increases the risk of CHD events, supporting our hypothesis [29]. The relationship between AF and SSS is well-established, as attested by the co-occurrence of the two conditions in the so-called “tachy-brady syndrome”, the involvement of the sinoatrial node in the development of atrial tachycardias [30], and the presence of diffuse atrial remodeling in SSS patients [31]. Further, the risk of both SSS and AF is elevated in association with polymorphisms and mutations in the *HCN4* gene [32], which encodes the hyperpolarization-activated ion channel HCN4, key for spontaneous pacemaker activity. Similarly, HF can result in dysfunction of the sinoatrial node and development of SSS [33], though no clearly described mechanism explains the increased risk of HF in individuals with SSS in our study. Reverse causation could be partly responsible: in a previous analysis of the ARIC and CHS cohorts, we found that higher levels of NTproBNP–a biomarker of volume overload and heart failure severity–were strongly associated with the subsequent development of SSS [4]. Future research should investigate these mechanisms in order to inform the management of SSS patients.

We observed that SSS patients receiving a pacemaker had a smaller increase of AF and HF risk that those without a device. These findings, however, need to be carefully interpreted given the lack of adequate information on patients' characteristics and, therefore, the potential for confounding by indication (i.e. healthier SSS patients being more likely to receive a pacemaker than those with more comorbidities).

Our study has relevant clinical and public health implications. First, the increased mortality and CVD risk in SSS patients could inform future clinical trials to refine the criteria for SSS treatment, for example evaluating a lower clinical threshold for pacemaker implantation or optimizing pacing modality, which may reduce risk of AF. Second, the higher risk of AF among SSS patients emphasizes the need for increased AF surveillance in this group. Additional studies should assess whether screening for AF in SSS patients could lead to better outcomes by providing adequate anticoagulation, rate, and rhythm control. And, third, because SSS is associated with increased CVD risk, guidelines for the management of SSS patients may need to address comprehensive cardiovascular prevention in addition to symptom relief, as well as a complete evaluation to identify possible substrates of SSS.

Limitations of this study include the partial information on SSS characteristics (such as severity of signs and symptoms), unavailability of outpatient data, where some SSS diagnoses might have been made, lack of detailed data on the management of SSS patients (e.g. type of pacemaker implanted), and the absence of systematic information on medication use at the time of SSS diagnosis and afterwards, which may influence the risk of the studied outcomes. Additionally, the small number of SSS events precluded the study of race, sex, or age differences. We also noticed significant between-cohort heterogeneity for the association of SSS with mortality and some outcomes, which may be due to differences in the age distribution of the cohorts. End-point ascertainment had also some shortcomings. Our definition of CHD included only hard end-points (myocardial infarction and definite coronary death) and, therefore, we could not assess the association of SSS with angina or subclinical CHD. In the ARIC cohort, heart failure was defined based on the presence of ICD codes in the discharge summary or death certificate, which could lead to overascertainment of events [34]. Nonetheless, study strengths include the large number of CVD events in these two community-based populations, the sociodemographic diversity of the study cohorts, the detailed data on cardiovascular risk factors, and the careful ascertainment and adjudication of cardiovascular outcomes and other covariates.

## Conclusion

We have shown that individuals who develop SSS are at higher risk of several cardiovascular complications than those without SSS. Our study should provide renewed impetus to understand the pathophysiology of SSS and improve the management of these patients.

## Supporting Information

Table S1 **Hazard ratios (95% confidence intervals) of mortality and selected cardiovascular diseases comparing individuals with and without sick sinus syndrome (SSS), using alternative SSS definitions, Atherosclerosis Risk in Communities (ARIC) study, 1987–2009.** Model 1: Cox proportional hazards model adjusted for age, sex, race, study center, education, smoking, body mass index, hypertension, total cholesterol, HDL cholesterol, diabetes, prevalent coronary heart disease, prevalent heart failure, and prevalent stroke. Model 2: As model 1, additionally adjusted for nonfatal incident coronary heart disease, incident heart failure, incident stroke and incident atrial fibrillation as time-dependent covariates
(DOCX)
