# Supplementary material for: Association of Sick Sinus Syndrome with Incident Cardiovascular Disease and Mortality: The Atherosclerosis Risk in Communities Study and Cardiovascular Health Study
Source: PLoS One. 2014 Oct 6;9(10):e109662. doi: 10.1371/journal.pone.0109662 (PMC4186847; doi:10.1371/journal.pone.0109662)
Supplement: Table S1 — Hazard ratios (95% confidence intervals) of mortality and selected cardiovascular diseases comparing individuals with and without sick sinus syndrome (SSS), using alternative SSS definitions, Atherosclerosis Risk in Communities (ARIC) study, 1987–2009. Model 1: Cox proportional hazards model adjusted for age, sex, race, study center, education, smoking, body mass index, hypertension, total cholesterol, HDL cholesterol, diabetes, prevalent coronary heart disease, prevalent heart failure, and prevalent stroke. Model 2: As model 1, additionally adjusted for nonfatal incident coronary heart disease, incident heart failure, incident stroke and incident atrial fibrillation as time-dependent covariates (DOCX) [file pone.0109662.s001.docx]

**Supplementary Table S1**. Hazard ratios (95% confidence intervals) of mortality and selected cardiovascular diseases comparing individuals with and without sick sinus syndrome (SSS), using alternative SSS definitions, Atherosclerosis Risk in Communities (ARIC) study, 1987-2009. Model 1: Cox proportional hazards model adjusted for age, sex, race, study center, education, smoking, body mass index, hypertension, total cholesterol, HDL cholesterol, diabetes, prevalent coronary heart disease, prevalent heart failure, and prevalent stroke. Model 2: As model 1, additionally adjusted for nonfatal incident coronary heart disease, incident heart failure, incident stroke and incident atrial fibrillation as time-dependent covariates

|  | Only validated SSS | | Validated SSS + ICD9 427.81 code without available charts | |
| --- | --- | --- | --- | --- |
|  | HR | 95% CI | HR | 95% CI |
| Mortality | | | | |
| Model 1 | 1.89 | 1.40-2.56 | 1.84 | 1.45-2.34 |
| Model 2 | 0.78 | 0.57-1.06 | 0.75 | 0.59-0.95 |
| Coronary heart disease | | | | |
| Model 1 | 1.40 | 0.66-2.94 | 1.63 | 0.94-2.82 |
| Model 2 | 0.58 | 0.27-1.24 | 0.67 | 0.38-1.17 |
| Stroke | | | | |
| Model 1 | 1.78 | 0.84-3.75 | 1.31 | 0.68-2.54 |
| Model 2 | 0.92 | 0.43-1.97 | 0.72 | 0.37-1.41 |
| Heart failure | | | | |
| Model 1 | 2.88 | 1.80-4.58 | 2.51 | 1.71-3.68 |
| Model 2 | 1.96 | 1.23-3.15 | 1.72 | 1.17-2.53 |
| Atrial fibrillation | | | | |
| Model 1 | 9.07 | 6.34-13.0 | 6.28 | 4.64-8.50 |
| Model 2 | 7.74 | 5.40-11.1 | 3.71 | 2.73-5.04 |
| Pacemaker implantation | | | | |
| Model 1 | 23.5 | 17.6-31.7 | 31.4 | 24.0-40.9 |
| Model 2 | 16.2 | 11.5-22.7 | 25.4 | 18.9-34.3 |
| CI: Confidence interval; HR: Hazard ratio; ICD9: International Classification of Disease 9^th^ edition; SSS: Sick sinus syndrome | | | | |
